# Microplastics in eviscerated flesh and excised organs of dried fish

**DOI:** 10.1038/s41598-017-05828-6

**Published:** 2017-07-14

**Authors:** Ali Karami, Abolfazl Golieskardi, Yu Bin Ho, Vincent Larat, Babak Salamatinia

**Affiliations:** 1 Laboratory of Aquatic Toxicology, Department of Environmental and Occupational Health, Faculty of Medicine and Health Sciences, Universiti Putra Malaysia, 43400 Selangor, Malaysia; 2 Discipline of Chemical Engineering, School of Engineering, Monash University Malaysia, 47500 Selangor, Malaysia; 3 HORIBA Jobin Yvon S.A.S., 231, rue de Lille, 59650 Villeneuve d’Ascq, France

## Abstract

There is a paucity of information about the occurrence of microplastics (MPs) in edible fish tissues. Here, we investigated the potential presence of MPs in the excised organs (viscera and gills) and eviscerated flesh (whole fish excluding the viscera and gills) of four commonly consumed dried fish species (n = 30 per species). The MP chemical composition was then determined using micro-Raman spectroscopy and elemental analysis with energy-dispersive X-ray spectroscopy (EDX). Out of 61 isolated particles, 59.0% were plastic polymers, 21.3% were pigment particles, 6.55% were non-plastic items (i.e. cellulose or actinolite), while 13.1% remained unidentified. The level of heavy metals on MPs or pigment particles were below the detection limit. Surprisingly, in two species, the eviscerated flesh contained higher MP loads than the excised organs, which highlights that evisceration does not necessarily eliminate the risk of MP intake by consumers. Future studies are encouraged to quantify anthropogenic particle loads in edible fish tissues.

## Introduction

Worldwide plastic production was estimated to reach 322 million metric tons in 2015^1^ whereby 5 to 13 million metric tons were reported to be disposed into the marine environment annually^2^. The plastics dumped in the environment may never completely degrade^3^ but instead fragment into smaller particles called microplastics (MPs), sized between 1 and 1000 µm^4^. The widespread distribution of MPs in aquatic bodies [e.g.,^5, 6^] has subsequently contaminated a diverse range of aquatic biota including those sold for human consumption such as fish^7^ and mussels^8^. Therefore, seafood products could be a major route of human exposure to MPs. For example, it was estimated that top European shellfish consumers might take up to 11,000 MPs per annum^8^. Microplastics were suggested to exert their harmful effects by providing a medium to facilitate the transport of other toxic compounds such as heavy metals^9^ and persistent organic pollutants (POPs)^10^ to the body of organisms. Upon ingestion, these chemicals may be released and cause toxicity^11^.

Dried fish are considered low-cost protein sources in many developing countries^12^. The purpose of the drying process is to create a desirable flavor and texture and/or to increase the shelf life by reducing the moisture content^13^. So far, nothing is known about the occurrence of MPs in dried fish that are intended for direct human consumption. Dried fish are often processed without any cleaning process, and although evisceration prior to drying helps to reduce bacterial contamination in fish^14^, this is not practical to many small fish species such as anchovies. Subsequently, this could potentially increase the chance of anthropogenic particle exposure to consumers. In this study, we investigated the potential presence of anthropogenic particles (MP and pigment particles) in the eviscerated flesh and excised organs of Indian mackerel (*Rastrelliger kanagurta*), spotty-face anchovy (*Stolephorus waitei*), greenback mullet (*Chelon subviridis*), and belanger’s croaker (*Johnius belangerii*). These species were chosen since they are often caught from the coastal waters of many Asian countries as well as in some other parts of the world^15–18^.

In aquatic organisms, the gills are the first organ exposed to anthropogenic particles during respiration^19, 20^, which increases the possibility that these particles can become stuck among the gill filaments. For example, following the exposure to high-density polyethylene (HDPE) fragments, these particles became trapped on the gills of the blue mussel (*Mytilus edulis*)^21^. Laboratory studies have later shown that MPs were able to be translocated into other tissues of fish^22, 23^. Most field studies on fish have investigated the occurrence of MPs in the gastrointestinal tract [e.g.,^24–26^] but little is known about their presence in their edible tissues.

Here, we investigated MP (0.001–1 mm), mesoplastic (1–10 mm), and macroplastic (>10 mm) loads and morphology (fragments, films, filaments, beads, and foams^27^) in the viscera and gills (hereafter are called excised organs) and eviscerated flesh of 4 dried fish species. All the isolated particles were initially sampled based on their similar density and morphology to MPs and then analyzed for their chemical composition using micro-Raman spectroscopy. Finally, to investigate if the extracted MPs contained hazardous inorganic substances, we further assessed the atomic composition of MP particles using field emission scanning electron microscopy (FESEM) equipped with an energy-dispersive X-ray spectroscopy (EDX). The results of this study will help to understand if removing viscera and gills could mitigate the intake of anthropogenic particles by consumers.

## Results

**Figure 1:**
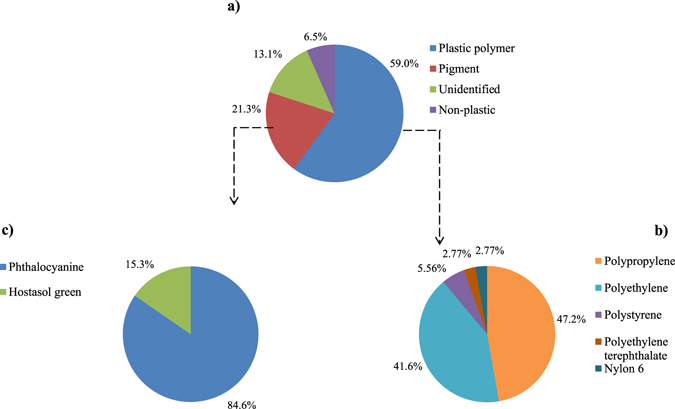
Particle compositions. (**a**) Pie chart of the percentage and chemical composition of the extracted particles from dried fish samples and their corresponding proportion of (**b**) plastic polymers, and (**c**) pigments.

**Figure 2:**
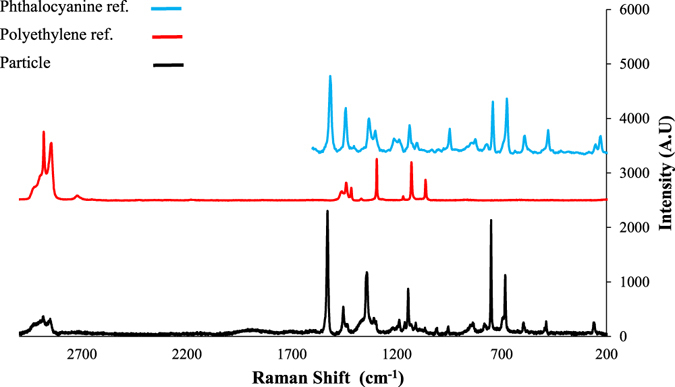
Raman spectrum of a particle identified as polyethylene + phthalocyanine and spectra of the reference materials.

**Figure 3:**
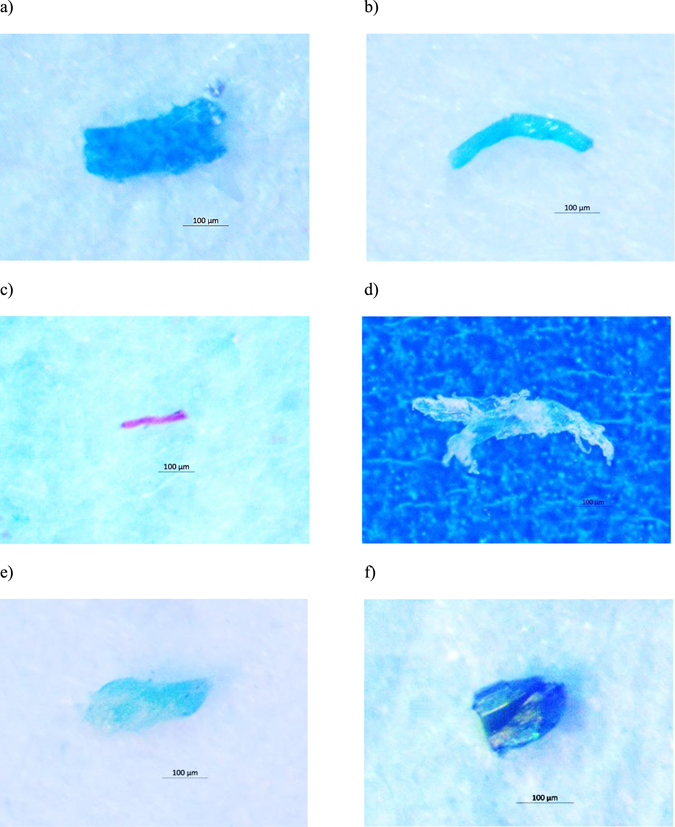
Isolated particles from dried fish. These particles were identified using micro-Raman spectroscopy as (**a**) Phthalocyanine, (**b**) Polypropylene + Phthalocyanine, (**c**) Polyethylene terephthalate, (**d**) Polyethylene, (**e**) Hostasol green, and (**f**) Actinolite.

**Figure 4:**
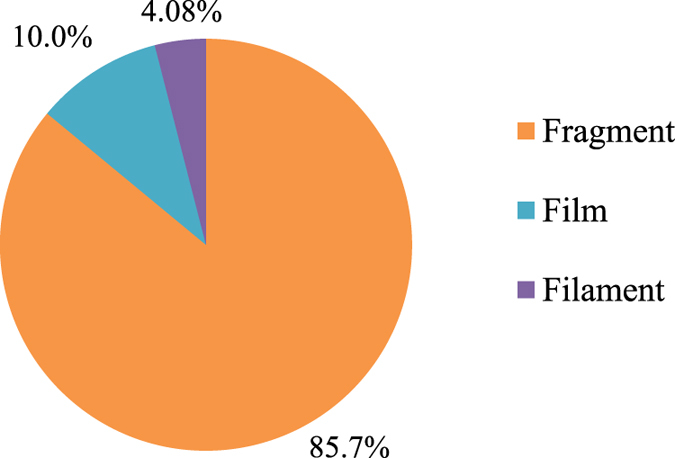
Pie chart of the morphology of isolated anthropogenic particles.

No particles were found in the procedural blanks. A total of 61 MP-like particles were isolated from the four dried fish species. As depicted by Fig. 1a, 36 particles (59.0%) were confirmed as MPs (i.e. particles confirmed as plastic polymer or plastic polymer plus pigment), 13 particles (21.3%) as pigments (i.e. particles confirmed as pigment), 4 particles (6.55%) were non-plastic items (i.e. cellulose or actinolite), and 8 particles (13.1%) remained unidentified. The most abundant plastic polymers were polypropylene (PP, 47.2%) followed by polyethylene (PE, 41.6%), polystyrene (PS, 5.56%), polyethylene terephthalate (PET, 2.77%), and nylon-6 (NY6, 2.77%) (Fig. 1b). Particles identified as pigments were phthalocyanine (84.6%) and hostasol green (15.3%) (Fig. 1c). Figure 2 shows the Raman spectra of a PE particle containing phthalocyanine. Figure 3a–f are the microscopic images of some of the isolated particles. With regards to morphology, the predominant type of anthropogenic particles were fragments (85.7%) followed by films (10.0%), and filaments (4.08%) (Fig. 4). No beads or foams were found among the samples.

**Figure 5:**
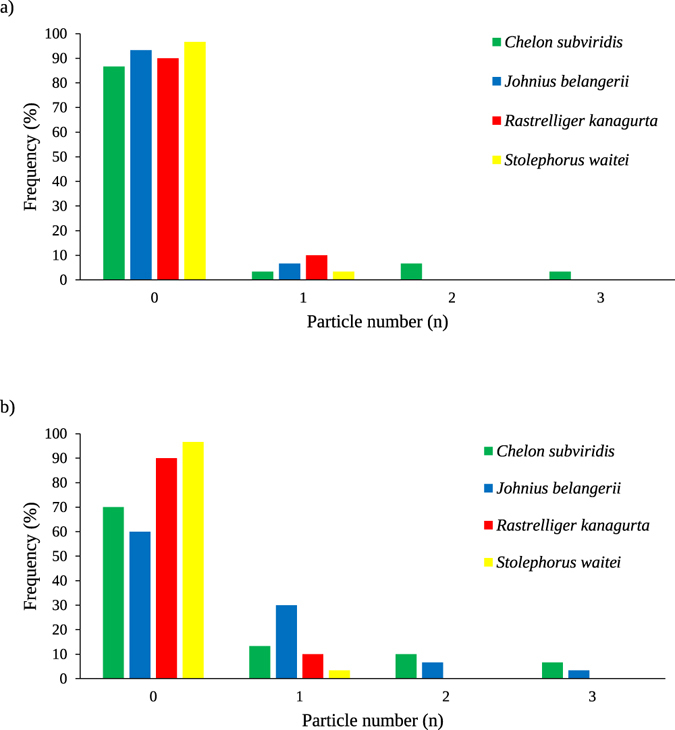
Frequency histograms of pigment and microplastic particles in the whole body (eviscerated flesh + excised organs) of tested dried fish species. Frequency histogram of (**a**) pigment and (**b**) microplastic particles in the whole body of *Chelon subviridis*, *Johnius belangerii*, *Rastrelliger kanagurta*, and *Stolephorus waitei*.

**Figure 6:**
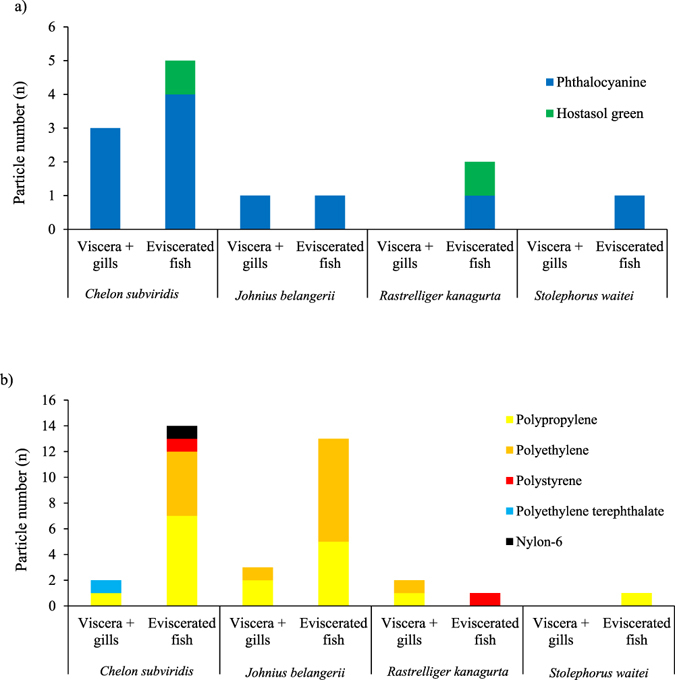
Stacked bar chart of the isolated particles from the excised organs or the eviscerated flesh. (**a**) The prevalence (n) of plastic polymer and (**b**) pigment particles; n = 30.

Between 0 and 3 pigments and MP particles were isolated from each individual fish. Figure 5a and b, present the frequency histograms of pigment and MP particle numbers across the tested species, respectively. Figure 6a and b are stacked bar charts of the number of extracted pigments and MP particles, respectively, isolated from the excised organs or the eviscerated flesh of each dried fish species. Surprisingly, 29 MPs and 9 pigment particles were isolated from the eviscerated flesh while 7 MPs and 4 pigment particles were from the excised organs. The abundance of anthropogenic particles per species ranged from 2 in *S. waitei* to 24 in *C. subviridis*. In *C. subviridis* and *J. belangerii*, Mann–Whitney *U* tests showed that the number of MPs in the eviscerated flesh was significantly higher than excised organs (*Z* = −2.43 and *Z* = −2.21, respectively; *p* < 0.05). No significant differences were, however, indicated in the number of pigment particles between the eviscerated flesh and excised organs. In *R. kanagurta* and *S. waitei*, the number of pigment particles or MP particles was comparable (*p* > 0.05) between the eviscerated flesh and excised organs.

There was a significant difference in the number of MPs (Kruskal-Wallis, *H* = 15.7, df = 3, *p* < 0.01) isolated from eviscerated flesh among the dried fish species. Dunn’s multiple comparison tests found higher MP loads in the eviscerated flesh of *C. subviridis* and *J. belangerii* than *R. kanagurta* and *S. waitei*. However, the number of pigment or MP particles in the excised organs was comparable among the fish species. Similarly, no significant difference, however, was noticed in the load of pigment particles extracted from eviscerated flesh among the species. Among the 5 identified plastic polymers, the prevalence of PP (Kruskal-Wallis, *H* = 8.56, df = 3, *p* < 0.05) and PE (Kruskal-Wallis, *H* = 9.14, df = 3, *p* < 0.05) were significantly higher in *C. subviridis* and *J. belangerii* than *R. kanagurta* and *S. waitei* while the concentration of other plastic polymers were comparable among all the tested species. Elemental analysis of the particles showed that all the anthropogenic particles contained carbon (C) and oxygen (O) while a few had nitrogen (N), chlorine (Cl) and sodium (Na) as well (Supplementary Information Fig. 1).

## Discussion

The ability of MPs to translocate from the digestive systems into other tissues of aquatic organisms^22, 23, 28^ has raised concerns about the safety of seafood products. Most of the studies on wild fish have assessed MP loads in the digestive tract while little has been done to investigate the presence of MPs in the edible fish tissues.

The absence of particles in the procedural blanks ensured the reliability of contamination prevention procedure employed by this study. Approximately one-fifth (21.3%) of the recovered particles were identified as pigments (phthalocyanine or hostasol green) owing to the strong Raman spectra of these additives. Recent studies have indicated that additives could complicate the identification of the chemicals within the samples [e.g.,^29^]. These synthetic pigments are extensively employed during the manufacturing of different materials, including plastics^30–33^. Initially, we suspected that these particles could be dyes. However, this hypothesis was rejected since none of the isolated particles shared the main characteristics associated with dyes (i.e. brittleness^34^). Some earlier studies inferred that pigment particles were plastics [e.g.,^35^] while others only suspected these particles to be plastics^8, 33^. Similar to the approach followed by the latter studies, we ensured that the pigment particles had an anthropogenic origin, but we could not confirm if they were in fact plastic polymers. One particle isolated from the excised organs of *R. kanagurta* was identified as actinolite. Actinolite is one of the 6 naturally occurring mineral silicate fibers that are referred to as asbestos, which has a wide application such as in textile, plastics, roofing, electrical insulation^36–38^.

Micro-Raman spectroscopy was unable to identify 13.1% of the isolated particles. The spectra of the samples collected from the field often differs with the spectra of pure materials, possibly due to the degradation process^39^. All the isolated particles in this study were sampled according to their similar density (i.e. having density <1.5 g/cm^3^) and morphology with MPs. Therefore, the unidentified particles are suspected to be MPs. The high occurrence of fragments in the fish could indicate their dominance in Malaysian coastal environments, which is consistent with fish caught in other regions of the world^23, 40^. Coastal areas have often been reported to be contaminated with MPs^41^, which is due to their vicinity to anthropogenic activities along the coast. The dominance of fragments in the tested fish in this study could reflect their prevalence in the water and sediments of Malaysian marine ecosystems. Consistent with our findings, Barasarathi *et al*.^42^ showed the dominance of fragments in the soils of a Malaysian mangrove forest. The absence of bead or foam microparticles in the eviscerated flesh or the excised organs of the tested fish could reflect their negligible prevalence in the natural environments.

Polypropylene and PE were the major recovered plastic polymers in the tested species, which is consistent with their massive production load and demand by various industries^1^. Consequently, this can lead to their broad distribution in the marine environment^43, 44^. Also, the lower density of PP (0.90–0.91 g/cm^3^) and PE (0.91–0.96 g/cm^3^) than seawater would cause them to float on the water surface. Over time, biofouling by micro- and macro-organisms have been suggested as a potential mechanism that could cause positively buoyant plastics to become less buoyant^45^ and, therefore, led to a more homogeneous distribution throughout the water column.

Surprisingly, in *C. subviridis* and *J. belangerii*, the MP load was significantly higher in the eviscerated flesh than excised organs. Initially, it was hypothesized that the fish might have been contaminated during handling on the fishing vessels or during the salting and drying processes. Interestingly, our recent study have shown the occurrence of MPs in most of the tested sea and lake salts produced in different countries^46^. However, this hypothesis was rejected because the fish were gutted in the laboratory after rinsing the body surface with water and ethanol (see *Contamination control*). Alternatively, the particles found in the eviscerated flesh could have been translocated from the alimentary tract. Several laboratory-based studies on fish have shown the translocation of MPs from the digestive system into other organs. For example, PE and PS particles (sizes: 200–600 µm) translocated from the stomach to the liver of flathead grey mullet (*Mugil cephalus*)^23^. In another study on zebrafish (*Danio rerio*), waterborne exposure to PS microspheres resulted in their accumulation in the gills and liver^22^. An earlier study in rats demonstrated the translocation of PS particles from the gut into the lymph^47^. Uptake through the layer of Peyer’s Patches from M-cells located within the small intestinal lymphoid tissue is a common route for the absorption of nano- and micro-particles^48^. M-cells are the differentiated epithelial cells with the ability to transcytosize macromolecules and particles^49^. Other mechanisms such as persorption might have been involved in the translocation of particles across the gastrointestinal mucosa^50^. The higher load of MPs in the eviscerated flesh of *C. subviridis* and *J. belangerii* highlights that evisceration does not fully eliminate the risk of MP uptake by consumers. Moreover, this study showed that quantifying MPs in the viscera may not truly represent their concentrations in the entire organism. Future studies are urged to assess MP loads, in not only the digestive tract, but also in the edible tissues of the fish. This strategy should better reflect the risks associated with the consumption of the fish caught from the natural environment.

In 2014, a total of 17 million tons of dried, smoked, or salted fish were produced for human consumption^51^. There is insufficient data, however, regarding the global consumption of dried fish. The species employed in this study were caught from Malaysian waters. Thus, consumers in neighboring countries could be exposed to the similar MP loads. Based on a report on the consumption of fish and fish products in the Asia Pacific region, the annual dried fish consumption in Bangladesh is 370 g/capita^52^. Considering the average fish weight (Table 1) and the number of anthropogenic particles (pigment + MP) per individual fish (between 0 and 3), consumers of *S. waitei* are expected to ingest between 0 to 246 MPs per annum. Similarly, consumers of *C. subviridis* (containing between 0 and 3 anthropogenic particles per fish), *J. belangerii* (containing between 0 and 3 anthropogenic particles per fish), or *R. kanagurta* (0 and 1 MP particles per fish) could ingest 0–68, 0–44, and 0–6 anthropogenic particles per annum, respectively. However, the majority of the tested fish in this study did not contain MP (Fig. 5b). Therefore, it is less likely that an individual would ingest the suggested maximum number of MPs per annum. Previous studies have shown that MPs adsorb POPs from the surrounding environment^53^ or contain additives that were incorporated into them during the manufacturing process^54^. Subsequently, these chemicals may desorb from the particles into the body of organisms upon ingestion^55^. However, recent studies have shown the intake of POPs by aquatic organisms from water and food exceeded the potential transfer of POPs from ingested MPs^56, 57^. According to the results of this study, the undetectable level of toxic heavy metals on the isolated particles does not support their potential toxicity and the mechanisms whereby MPs cause toxicity are still unclear. Therefore, despite the potential for a maximum of 246 anthropogenic particles to be ingested by a human per year, we cannot evaluate the health risks associated with the consumption of dried fish at this moment. The increase in plastics disposal^2^ coupled with their continuous fragmentation^58^, is expected to increase MPs concentrations over time. As such, it will become increasingly important to regularly assess MP loads in seafood products, including dried fish.

**Table 1:** Average total weight and length ± SD of the dried fish used in this study. Number of fish examined per species (n) = 30.

Common name	Species	Total weight (g)	Total length (cm)
Greenback mullet	*Chelon subviridis*	16.3 ± 1.812	12.7 ± 0.4460
Belanger’s croaker	*Johnius belangerii*	25.2 ± 0.381	13.6 ± 0.2160
Indian mackerel	*Rastrelliger kanagurta*	58.5 ± 3.755	18.6 ± 0.2687
Spotty-face anchovy	*Stolephorus waitei*	1.50 ± 0.1568	6.66 ± 0.4440

Given the fact that dried fish are often consumed as a whole, they may be responsible for the translocation of a significant amount of MPs into the body of consumers. Higher MP loads in the edible tissues than excised organs of two dried fish species indicates that removing gills and viscera does not necessarily reduce MP uptake by consumers of dried fish. The results of this study underscores the importance of assessing edible fish tissues for MP presence and to better understand the ability of MPs to translocate into other organs.

## Methods

### Materials and chemicals

Packed dried *C. subviridis*, *J. belangerii*, *R*. *kanagurta*, and *S*. *waitei* were purchased from local markets in Malaysia (Table 1). Sodium iodide (NaI), potassium hydroxide (KOH), and ethanol 95% were purchased from R&M Chemicals (UK). Solutions of NaI (4.4 M) and KOH (10% w/v) were prepared by dissolving the powder/pellet in ultrapure deionized water. The GF/D microfiber filter membranes (pore size 2.7 μm) and filter membranes No. 540 (hardened ashless, pore size 8 μm) were supplied by Whatman. The 149 μm-pore size filter membranes were purchased from Spectrum Laboratories (USA).

### Sample preparation

Each fish was placed on pre-cleaned aluminum foil, and the total length and weight was recorded. An equal number of fish per pack (3–6 packs per species) was used to provide a total number of 30 fish for each species (n = 30). The gill arches were carefully removed by cutting through the bone at the top and bottom where the gills joined the head. The viscera was removed by cutting the fish beginning at the vent and continuing to the throat. Gills and viscera (excised organs) were placed together into a 250 mL DURAN laboratory glass bottle sealed with a premium cap and pouring ring (Schott, Germany). The eviscerated flesh (fish without gills and viscera) was placed into a separate 250 mL laboratory bottle and were subjected to digestion.

### Microplastic isolation

Microplastic isolation from the eviscerated flesh and excised organs of dried fish were done according to the method of Karami *et al*.^59^. Briefly, 200 mL of KOH (10% w/v) was added to each bottle containing either excised organs, or the eviscerated flesh, and were incubated at 40 °C for 72 h. The digestate was then vacuum filtered through a 149 μm filter membrane. To separate the high-density particles (i.e. bone fragments and scales), the filter membrane was soaked in 10–15 mL of 4.4 M NaI (density: 1.5 g/mL), sonicated at 50 Hz for 5 min and agitated on an orbital shaker at 200 rpm for 5 min. Eventually, the solution was centrifuged at 500 × g for 2 min, and the supernatant containing MPs was vacuum filtered through another filter membrane (pore size 8 μm). This process was repeated once more to ensure complete isolation of MPs.

### Visual identification

Microscopical examination of the filter membranes was performed using a Motic SMZ-140 stereomicroscope (Motic, China). Particles resembling MPs were sampled based on their morphological characteristics, such as color and shape, as explained by Karami *et al*.^46^ Fragments (irregular shape with uneven surface), fibers/filaments (thin and elongated), beads (spherical and ovoid), films (thin plane of flappy), or foams (lightweight and highly porous). Selected particles were photographed using a camera (AxioCam, ERc 5S, Germany) coupled with a microscope.

### Raman spectroscopy and FESEM-EDX analyses

Particles were analyzed over a range of 150 to 3000 cm^−1^ using a Raman spectrometer (Horiba LabRam HR Evolution) equipped with a Single Mode Open Beam Laser Diode (Innovative Photonic Solutions) operating at a wavelength of 785 nm coupled with a charge-coupled device detector (Horiba Synapse). Before the library search, to reduce noise and enhance the spectrum quality without losing subtle spectral information, each spectrum passed through a baseline correction and denoising procedure (Labspec 6, Horiba Scientific). Pre-processed spectra were then evaluated and compared to the following spectral libraries: Raman polymers and monomers from Bio-Rad Sadtler and Raman Forensic from Horiba using the KnowItAll software from Bio-Rad. The Correlation algorithm (KnowItAll, Bio-Rad) was used to evaluate each query spectrum to the spectra of the databases. The Hit Quality Index (HQI) was used to rank the results of the spectral search. To assess the inorganic composition of isolated MPs, all particles identified as plastic polymers were examined using a FESEM (Hitachi Ultra-high resolution SU8010) operating at 5 keV and equipped with an Oxford-Horiba Inca XMax50 energy-dispersive X-ray (EDX; Oxford Instruments Analytical, High Wycombe, England). The detection limit of the machine was around 1000 pg/µg for most of the heavy metals.

### Contamination control

To minimize contamination, cotton lab coat, nitrile gloves were worn at all times. All the liquids (deionized water, ethanol, KOH, and NaI) were filtered through a GF/D microfiber filter membrane (pore size 2.7 μm). The glassware and instruments, such as dissecting scissor and forceps, were washed once with dishwashing liquid, rinsed with deionized water, and finally with ethanol. To remove any potential particles attached to the fish body surface, the outer part of the fish was rinsed twice with ultrapure deionized water and once with ethanol. The work surface was pre-cleaned with 70% ethanol every time before dissection. The entire procedure was carried out in a horizontal laminar flow cabinet (AHC-4A1-ESCO) to avoid potential contamination with airborne MPs. To monitor and correct potential contamination, one procedural blank with 10% KOH was tested simultaneously during the isolation procedure, and another procedural blank containing NaI solution was tested simultaneously during the density separation process.

### Statistics

Shapiro–Wilk test was used to assess normality of the data. Upon data transformations, normal distribution was not achieved. Therefore, the Mann–Whitney *U* test was used to compare pigment or MP particle loads between the eviscerated flesh and excised organs of each fish species. A Kruskal–Wallis test (non-parametric one-way analysis of variance) was employed to compare the number of extracted pigments or MP particles in the excised organs or eviscerated flesh among the tested species. Also, the concentration of each polymer (PP, PE, PS, NY6, PET) was compared among the species with a Kruskal–Wallis test. The analysis was followed by using Dunn’s multiple comparison tests if a significant difference (P < 0.05) was obtained. Statistical analyses were performed using SPSS (IBM SPSS Statistics V. 24).

## Electronic supplementary material

Supplementary Information
