# Supplementary material for: Microplastics in eviscerated flesh and excised organs of dried fish
Source: Sci Rep. 2017 Jul 14;7:5473. doi: 10.1038/s41598-017-05828-6 (PMC5511207; doi:10.1038/s41598-017-05828-6)
Supplement: Supplementary file 1 — Supplementary Information [file 41598_2017_5828_MOESM1_ESM.pdf]

Supplementary Information

Microplastics in eviscerated flesh and excised organs of dried fish

Ali Karami[*,1], Abolfazl Golieskardi[1], Yu Bin Ho[1], Vincent Larat[3], Babak Salamatinia[2]

Figure 1. **Field emission scanning electron microscopy (FESEM) image and energy-dispersive X-ray (EDX) spectra of some of the extracted microplastics**. Field emission scanning electron microscopy image of one a) polyethylene, b) polystyrene, and c) polypropylene particle. Energy-dispersive X-ray spectra show d) and e) carbon (C) and oxygen (O) content, and f) C, O, nitrogen (N), sodium (Na), and chlorine (Cl) contents.

d)

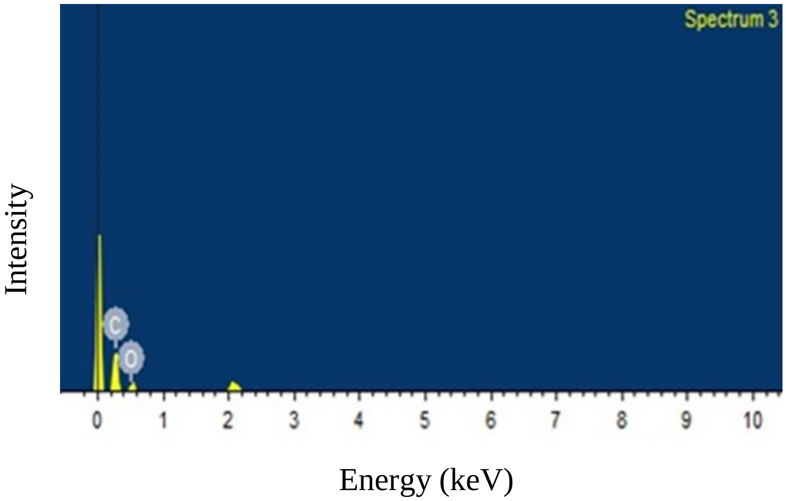

a)

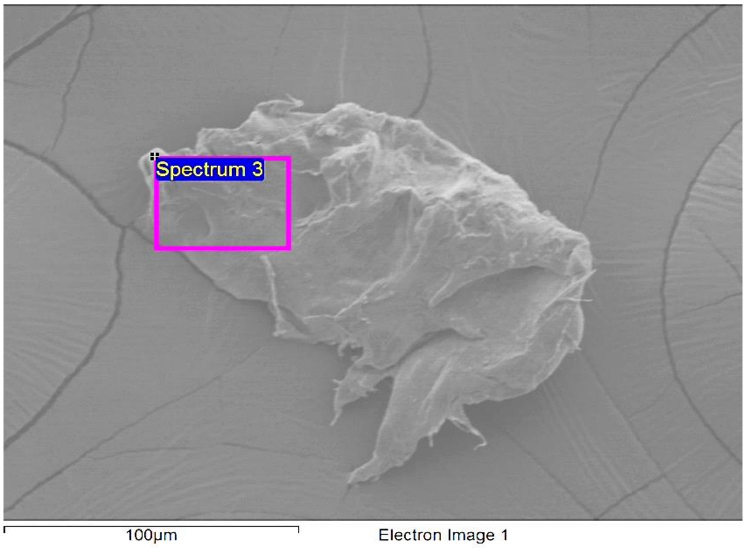

e)

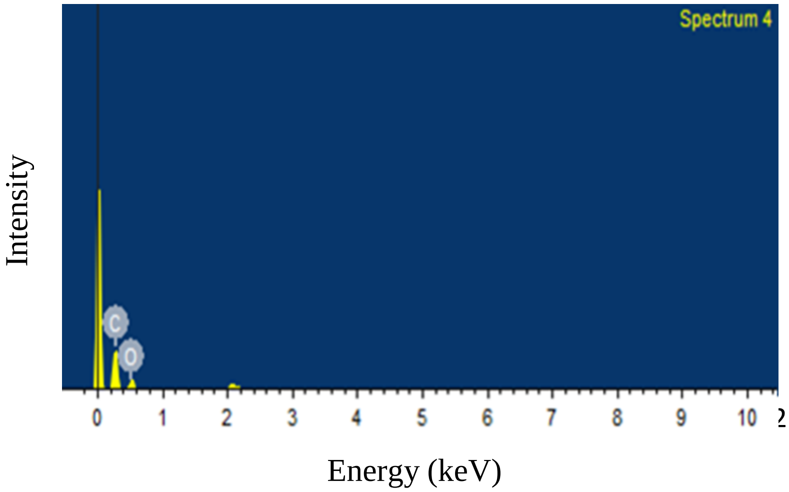

b)

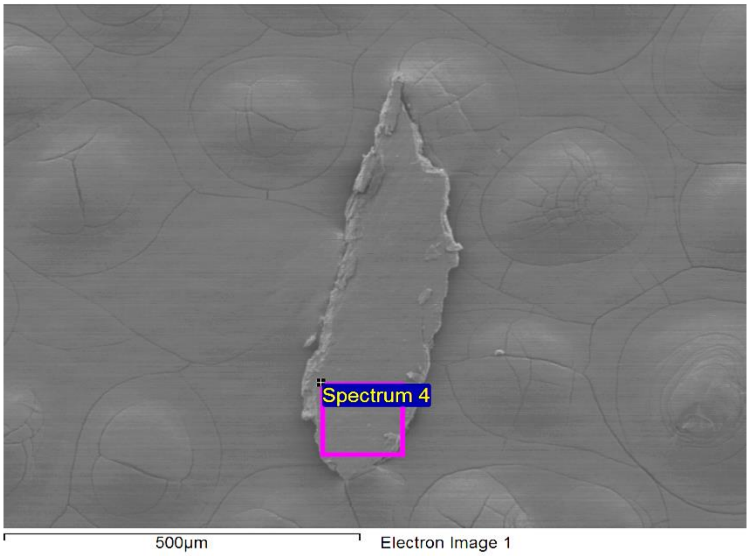

f)

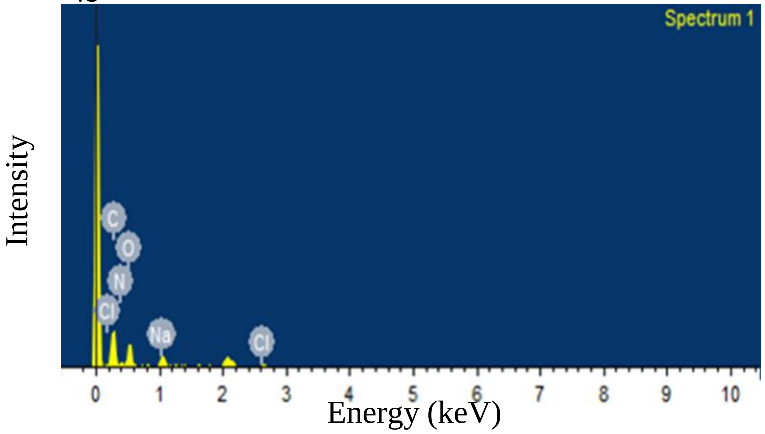
Spectrum 1
C
O
N
Cl
Na
Cl
0 1 2 3 4 5 6 7 8 9 10
Intensity
Energy (keV)

c)

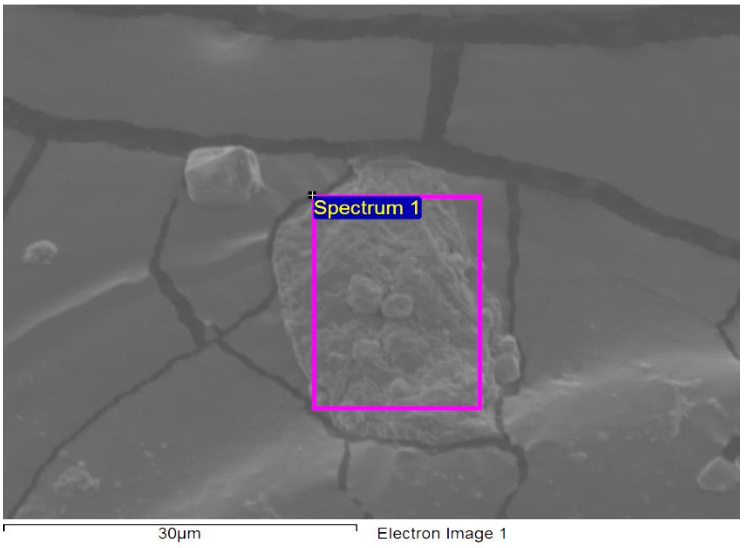
Spectrum 1
30µm
Electron Image 1
